# Narrative review of the impact on physicians of administering euthanasia or physician-assisted suicide and its association with moral distress

**DOI:** 10.1017/S1478951525000240

**Published:** 2025-06-03

**Authors:** Priyanka Pinto, Gerald Blaise Fogarty, David Kissane

**Affiliations:** 1 School of Medicine, University of Notre Dame Australia, Darlinghurst, NSW, Australia; 2 Cabrini Health, Malvern, VIC, Australia; 3 Department of Psychiatry, Monash University, Clayton, VIC, Australia

**Keywords:** Moral distress, euthanasia, medical aid in dying, physician-assisted suicide, palliative care

## Abstract

**Background:**

Moral distress affects a significant proportion of clinicians who have received requests and participated in euthanasia or physician-assisted suicide (E/PAS) globally. It has been reported that personal and professional support needs are often unaddressed, with only a minority of those reporting adverse impacts seeking support.

**Objectives:**

This study aimed to review studies from 2017 to 2023 for the perceived risks, harms, and benefits to doctors of administering E/PAS and the ethical implications for the profession of medicine resulting from this practice.

**Methods:**

The search explored original research papers published in peer-reviewed English language literature between June 2017 and December 2023 to extend prior reviews. This included both studies reporting quantitative and qualitative data, with a specific focus on the impact on, or response from, physicians to their participation in E/PAS. The quantitative review was guided by the Preferred Reporting Items for Systematic Reviews and Meta-Analyses (PRISMA). The qualitative review used the Critical Appraisal Skills Programme to assess whether studies were valid, reliable, and trustworthy.

**Results:**

Thirty studies (quantitative *n* = 5, qualitative *n* = 22, mixed methods *n* = 3) were identified and fulfilled acceptable research assessment criteria. The following 5 themes arose from the synthesis of qualitative studies: (1) experience of the request prior to administration; (2) the doctor’s role and agency in the death of a patient; (3) moral distress post-administration; (4) workload and burnout; and (5) professional guidance and support. Both quantitative and qualitative studies showed a significant proportion of clinicians (45.8–80%) have been adversely affected by their involvement in E/PAS, with only a minority of those reporting adverse impacts seeking support.

**Significance of results:**

Participation in E/PAS can reward some and cause moral distress in others. For many clinicians, this can include significant adverse personal and professional consequences, thereby impacting the medical profession as a whole.

## Introduction

The legalization of euthanasia or physician-assisted suicide (E/PAS) or voluntary assisted dying (VAD) in Australia in recent years has created debate on the various ethical, moral, and psychological repercussions on patients and healthcare workers in end-of-life care. Euthanasia is where “a physician, actively and intentionally ends a patient’s life by some medical means such as a lethal injection” (Emanuel et al. 2016, 80). Whereas physician-assisted suicide is defined as “a physician providing medications or other interventions to a patient with the understanding that the patient intends to use them to commit suicide” (80). For further clarity, VAD is the practice of E/PAS in Australia and MAiD (medical assistance in dying) is the practice of E/PAS in Canada. Some physicians support and perceive benefit in patient care through E/PAS; others oppose and hold concern for harm. However, the extant research encompasses patient and physician attitudes toward E/PAS rather than reporting actual experiences of those who have administered E/PAS. Kelly et al. (2020) sought to fill this gap in the literature with their review of findings about moral distress published until 2018. This review is a continuation by examining findings on the moral and psychological effects of administering E/PAS on physicians globally in studies ranging from 2017 to 2023.

Moral distress has been defined as “distress that arises when one knows the right thing to do, but institutional constraints make it nearly impossible to pursue the right course of action” (Jameton 1984, cited in Banerjee and Alici 2021, 6). This can occur especially if doctors feel that they have caused harm to a patient – more so if it is avoidable harm (Grassi et al. 2021). This can eventually lead to moral injury, which is the “lasting psychological, biological, spiritual, behavioural and social impact of perpetrating, failing to prevent, or bearing witness to acts that transgress deeply held moral beliefs and expectations” (Litz et al. 2009, 700 cited in Houle et al. 2024). Moral injury can also be understood as the longstanding effects of moral distress; “after which the providers’ moral integrity erodes and can result in desensitisation and withdrawal in the face of other moral aspects of care” (Banerjee and Alici 2021, 2). According to Čartolovni et al. (2021), “Moral injury involves a deep emotional wound … the concept of moral injury was considered under other concepts such as stress of conscience, regrets about an ethical situation, moral distress and ethical suffering, guilt without fault, and existential suffering with inflicting pain” (590). Thus, the literature makes clear that it is impossible for a physician to simply act as a “provider” without deeply experiencing the consequences of their actions and, in some cases, leading to detrimental impacts to their mental health. This is the case for both physicians in the initial stage of receiving a request for E/PAS and for those who have enacted E/PAS.

According to Wibisono et al. (2022), 52% of participants showed moral distress in response to a scenario about an assisted death request from patients. However, there is also evidence to show that a proportion of doctors do not experience moral distress after involvement with requests for E/PAS (Beuthin et al. 2020). In fact, Beuthin et al. (2020) demonstrated that physicians involved in E/PAS experienced an “intimate outpouring of gratitude by patients and families…[which] seemed to buoy, empower and affirm participants” (6). The common ground between physicians who do and do not experience moral distress after receiving requests and/or administering E/PAS appears to be that both desire to act in the best interest of the patient, including to uphold the principles of beneficence, non-maleficence, justice, and respect for autonomy. Many physicians, who may oppose E/PAS, are often not doing so out of religious convictions, ignorance, or indifference to their patients’ suffering (Van den Ende et al. 2022, 6). They may experience a genuine psychological conflict that is triggered by being asked to end life in a profession that hinges on saving and preserving life; *primum non nocere.* Given this background and the growth of countries permitting E/PAS, this study aimed to review systematically recent studies from 2017 to 2023 for the risks, harms, or benefits to doctors of administering E/PAS and the ethical implications for the profession of medicine resulting from this practice.

## Methods

### Eligibility criteria

#### Inclusion criteria

The search looked for original research papers published in peer-reviewed English language literature between June 2017 and December 2023. This included both studies reporting quantitative and qualitative data, with a specific focus on the impact on, or response from, physicians participating in E/PAS. The quantitative review was guided by the Preferred Reporting Items for Systematic Reviews and Meta-Analyses (PRISMA; Moher et al. 2009). The qualitative review used the Critical Appraisal Skills Programme (CASP 2013) to assess whether studies were valid, reliable, and trustworthy. The results from the qualitative and quantitative studies were summarized using a “best evidence” synthesis (Slavin 1995). This entailed a systematic approach to selection of papers and a descriptive approach to the narrative synthesis of findings.

#### Exclusion criteria

As this review focused on original research evidence regarding the impact of responding to requests and enactment of E/PAS, reports of findings from surveys solely focusing on physician views/opinions about E/PAS (rather than experiences of) were excluded, as were studies of physician experiences of end-of-life care in general. In Australia and most legislations, practitioners with decision-making responsibility for E/PAS are physicians, hence, studies focusing on moral distress in other healthcare workers have been excluded. In addition, where identified by the initial search terms, conference abstracts, commentaries or editorial papers were manually excluded.

### Search strategy

The following MeSH terms were used for the search: “euthanasia” or “voluntary assisted dying” or “assisted suicide” or “medical assistance in dying” or “hastened death” or “end of life.” In order to ensure a sufficiently comprehensive search of published data, broad terms were needed that reflected the relevant terminology in this field. Four databases were searched: PubMed, Medline, Embase, and PsycINFO. A stepwise process was undertaken with review of title, abstract, and then full paper where relevant.

### Data collection process

Potential papers were screened by the first author (PJP). Uncertainties were discussed with the other authors for resolution. As required by PRISMA (PRISMA; Moher et al. 2009) and CASP guidelines (CASP 2013), the following details were extracted from each relevant paper: study population, locality, outcome measures, and data analytic methods. Quality criteria were applied using accepted metrics for evaluation of qualitative research publications (O’Brien et al. 2014). A descriptive approach to summarizing key findings was necessarily derived from the systematic selection of published research findings. Our analysis involved grouping findings into initial codes, then forming these into categories, and ultimately themes, using a deductive, top-down analytical approach (Bengtsson 2016). The codes and emergent themes were discussed by the researchers to reconcile discrepancies and ensure trustworthiness and rigor.

## Results

**Figure 1.**
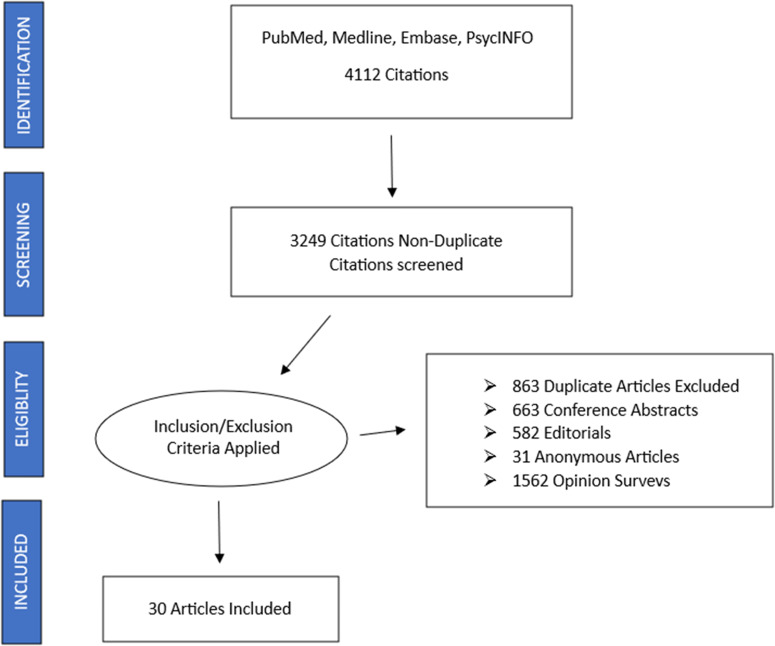
Process of study selection.

In total, the search resulted in 4112 papers being identified. The predominant reasons for excluding results were that they were conference abstracts or papers (*n* = 1044), editorials, commentaries, or ethical/legal perspectives (*n* = 582) or were written by an anonymous source (*n* = 31). The majority of the remaining studies (*n* = 1562) were research on opinions (general population surveys, surveys of health professionals’ views), or had a focus on end-of-life care in general rather than euthanasia/assisted suicide. Figure 1 depicts this process of study selection. Following the screening, 30 relevant papers were reviewed.

Of the 30 studies this paper focuses on, half (*n* = 15) come from North America, 20% (*n* = 6) from Australia, 10% (*n* = 3) from the Netherlands, and the remainder from single countries. Seven were reviews, with the majority employing qualitative methodology on samples ranging from 2 to 33 participants from General Practice, ICU, Palliative Care and Oncology, one survey of 1374 Dutch physicians and another of 150 Canadian intensive care doctors. All studies occurred between 2017 and 2023, reflecting the recent focus of this review. Given the predominance of qualitative reports, our analysis that follows is thematic in nature. Table 1 provides an overview of key studies exploring how physicians cope after participating in euthanasia or physician-assisted suicide.

**Table 1.** Key studies of physician coping after involvement in euthanasia or physician-assisted suicide

Author/s	Year	Country	Type of study	No. of participants	Method	Key findings
Emanuel et al.	2016	Netherland, Belgium, USA and Canada	Qualitative & quantitative	Systematic review	The published literature was searched from 1947 until 2016, with a focus on original data from surveys, jurisdictions that have legalized E/PAS and death certificate studies.	86% of physicians dread the emotional burden of E/PAS. Interviews of physicians who have participated in E/PAS indicate that the decision to go through with a procedure is neither easy nor straightforward.
Ten Cate et al.	2017	Netherlands	Qualitative	33	33 interviews were conducted with general practitioners (GPs) from various regions in the Netherlands.	Participants reported that they experienced tension when there was a lack of family support and a sense of not having used all the means to alleviate the patient’s pain.
Wang	2017	United States	Qualitative	2	A case study was conducted using a stepwise analysis of communication and actions between physicians and family.	This case highlights the significant distress experienced by each party. Hospitals require comprehensive protocols to handle complex ethical and psychosocial issues surrounding physician-assisted dying.
Khoshnood et al.	2018	Canada	Qualitative	16	Semi-structured interviews were conducted with 16 physicians. An inductive thematic analysis approach guided data collection.	Participants described workload and relationship challenges to providing MAiD including relational closeness creating ambivalence and role confusion.
Shaw et al.	2018	Canada	Qualitative	8	Interviews were audiorecorded, transcribed, and analyzed through qualitative thematic analysis.	Participants found providing MAiD satisfying. Emotional challenges related to providing MAiD included: having to deny MAiD to patients who did not qualify; disagreements with colleagues; dealing with the grief of the family.
Snijdewind et al.	2018	Netherlands	Qualitative	28	A secondary analysis of in-depth interviews was conducted with 28 Dutch physicians who had experience with a complex case of EAS.	Second requests for EAS based on non-medical reasons were more frequent and created ambivalence about criteria with patient pressure.
Bouthiller & Opatrny	2019	Canada	Qualitative	22	An exploratory qualitative study based on semi-structured interviews with 22 physicians. Thematic descriptive analysis was used.	The majority of physicians who refused to administer MAiD did not oppose it in theory. The reason most cited is not religious, but rather suggests an unavoidable psychological conflict involved in ending human life.
Brassfield et al.	2019	United States	Qualitative	1	A case was examined from Vermont in which a terminally ill patient requests aid-in-dying from her primary care physician.	The case reveals the conflict between respect for patient autonomy and a desire to uphold beneficence and non-maleficence as well as the moral distress that results from trying to act against one’s conscience.
De Boer et al.	2019	Netherlands	Qualitative	15	Semi-structured in-depth interviews were conducted with 15 GPs. Interviews were analyzed with the use of the framework method.	The majority of Dutch physicians experience pressure which caused moral distress when faced with a request. Interviews revealed 6 major categories: (1) Emotional blackmail, (2) Control by others, (3) Doubts about fulfilling criteria, (4) Counterpressure by patient’s relatives, (5) Time pressure, and (6) Organizational pressure.
Evenblij et al.	2019	Netherlands	Qualitative	1374	A cross-sectional survey was conducted among Dutch physicians. This study aimed to explore concerns, and pressure experienced by physicians who receive requests for E/PAS.	Of the 245 physicians who granted the EAS request, 66.7% reported comfortable feelings after having performed EAS. Simultaneously, 80.0% reported feelings of discomfort: 49.6% of the physicians experienced it as burdensome, 45.8% as a heavy responsibility and 44.2% as emotional. Overall, 62% of physicians did not seek support.
Gamondi et al.	2019	Switzerland	Qualitative	23	A qualitative study was conducted with 23 palliative care physicians across Switzerland. Thematic analysis was used to interpret data.	Many participants struggled to reconcile their understanding of good palliative care as providing comfort and relief (beneficence and non-maleficence) with patients’ wishes to exercise their autonomy.
Beuthin et al.	2020	Canada	Qualitative	8	A descriptive methodology and thematic analysis was used. Data were collected through audiotaped, semi-structured interviews in person or by phone.	Participants indicated that providing individuals with an assisted death generated an intimate outpouring of gratitude by patients and families, and the extent of this landed on the prescribers in an overwhelming and significant way.
Kelly et al.	2020	Australia	Qualitative & quantitative	Systematic review	Original research papers were identified reporting qualitative or quantitative data published in peer-reviewed literature between 1980 and March 2018. PRISMA and CASP guidelines were followed.	30–50% of doctors described an emotional burden or discomfort about participation, while findings also identified a satisfaction in believing the request of the patient was met. Significant, ongoing adverse personal impact was reported between 15% and 20%.
Koksvik	2020	Canada	Qualitative	9	In-depth interviews were carried out with 9 Quebec physicians. Two interviewers each independently analyzed the transcripts, employing an iterative and thematic approach.	The major themes of MAiD in relation to aggressive life-sustaining treatment, conscientious objection and uneven distribution of work.
						Findings expose a complexity and contentiousness within clinical practice, which remains under researched.
Bellolio & Rosso	2021	Chile	Quantitative	Systematic review	A systematic review was carried out for original articles indexed in the PubMed database.	The available studies indicate that the decriminalization of euthanasia has a negative impact on the professional work of physicians.
Mathews et al.	2021	Canada	Qualitative	23	This study aims to explore the experience of frontline palliative care providers about the impact of medical assistance in dying (MAiD) on palliative care practice.	Themes identified included impact on palliative care providers personally and on their relationships with patients; and consumption of palliative care resources to support assisted death.
McDougall et al.	2022	Australia	Qualitative	Systematic review	Exploration of experiences of junior doctors, incorporating quotations and a discussion of the ethical implications of conscientious objection to VAD.	JMOs are placed in a distressing and vulnerable position in regards to being at the receiving end of negative judgment from more senior doctors who disagree with their views and mediation between patient and family.
Rutherford et al.	2021	Australia	Qualitative	25	Semi-structured interviews with Victorian doctors with no in-principle objection to VAD were conducted. Interviews were recorded, transcribed, and analyzed using thematic analysis.	Doctors perceive or experience VAD to fundamentally challenge traditional medical practice.
						Barriers to access to VAD derive from applicant, communication, and willingness and availability of participating physicians.
Rutherford et al.	2021	Australia	Quantitative & qualitative	3	26 publications detailing 19 studies were identified. Thematic analysis of quantitative and qualitative findings was performed.	Findings revealed key themes including ‘Attitudes toward regulation’, and ‘Professional and personal impact of legalization’ describing tensions between palliative care and VAD.
St Ledger et al.	2021	Ireland	Qualitative	18	Eighteen senior and junior physicians involved in 21 patient case studies were interviewed. Interviews were transcribed verbatim and analyzed.	Junior residents reported most instances of moral distress, triggered by perceived futility, lack of continuity, protracted decisions and failure to ensure ‘good death.’
						Senior physicians’ triggers included constraint of clinical autonomy. Moral distress was far reaching, affecting personal life, working relationships and career choice.
Ward et al.	2021	Canada	Quantitative & qualitative	4	This study employed a scoping review: (1) identified research articles, (2) identified relevant studies, (3) select studies based on inclusion/exclusion criteria, (4) chart the data, and (5) summarize the results.	This scoping review found that HCPs experienced a variety of emotional responses to providing or providing support to MAiD. Some HCPs experienced positive emotions through helping patients at the end of the patient’s life. Still other HCPs experienced very intense and negative emotions such as immense internal moral conflict.
Anderson et al.	2022	Canada	Qualitative	150	English-speaking intensivists and critical care trainees were surveyed in 7 Canadian provinces. A qualitative descriptive framework was best suited for the data given the breadth of questions posed in the survey and the range of answers received.	Six broad themes emerged from our analysis of the data, reflecting areas of consensus as well as substantial disagreement between respondents. Moral distress related to end-of-life care was a common theme for participants. Some respondents stressed the importance of ensuring consensus within the care team and between the team and patient’s family to minimize moral distress.
Campbell et al.	2022	United States	Quantitative	8	An anonymous, multi-wave, survey (RR = 55%) was conducted with an enriched sample (*n* = 583) of Colorado physicians caring for potential MAiD patients.	Among MAiD consultants, 75% reported that their most recent MAiD case was professionally rewarding, though 75% also reported that it was time consuming and 46.9% reported that it was ethically challenging.
Dholakia et al.	2022	Canada	Qualitative	Systematic review	The aim of this study was to determine the emotional impact on HCPs involved in MAiD through a systematic review and qualitative meta-synthesis.	The thematic meta-synthesis showed 3 themes: (1) polarized emotions including moral distress (*n* = 153), (2) reflective emotions with MAiD (*n* = 251), and (3) professional value-driven emotions (*n* = 352).
Kortes-Miller & Durant	2022	Canada	Qualitative	23	One semi-structured focus group and one-to-one interviews transcribed and analyzed thematically.	Four themes were identified from the interviews: relationships, motivation, time and resources, and getting others on board, contributing to the burden of physicians as they work to provide MAiD.
Sellars et al.	2022	Australia	Qualitative	32	In-depth, semi-structured interviews were conducted with 32 Victorian doctors involved in VAD during the first 12 months since it became legal in Victoria.	Findings revealed a major tension in not just how doctors’ perceptions impacted their own well-being and satisfaction, but also how these challenged their continued involvement in VAD and the system’s overall ability to function.
Wibisono et al.	2022	Australia	Quantitative & qualitative	Systematic review	Articles were collected that met the criteria from databases and were compiled in a Microsoft Excel spreadsheet for data extraction.	12 of 18 studies focused on moral distress as the psychological implication of engagement with assisted death services, while the others focused on emotional pressure and burdening, negative feelings, frustration, apprehension, powerlessness, uncertainty, job leaving, professional burnout, feeling of discomfort, moral ambiguity, emotional pressure, and fear of social stigma.
Batzler et al.	2023	Germany	Quantitative	22	A questionnaire was designed by the study team. Data analysis was performed using Microsoft Excel 2020 and other programs.	As the perceived symptoms were regarded burdensome, moral concerns rarely occurred. HCP in this study stated that they generally approve VSED (E/PAS). At T1, the symptom complex of psychological distress, anxiety, and agitation was the most burdensome; at T2, it was thirst.
Kono et al.	2023	Japan	Qualitative	Systematic review	1,036 studies were retrieved from PubMed and EthxWeb and screened using exclusion criteria. The subject studies collected were analyzed qualitatively using a thematic analysis	Ambiguous requirements can confuse physicians when deciding whether to allow a patient to be euthanized. In some cases, GPs felt pressured by patients to grant E/PAS.42% of GPs in the Netherlands do not agree with ADEs and will not perform euthanasia as stated in ADE.
Wiebe & Kelly	2023	Canada	Quantitative & qualitative	23	Online survey with closed and open-ended questions about clinicians’ experiences with individual patients making track 2 MAiD requests.	The most common challenges providers reported were related to patients having concurrent mental illness, noted in 37 assessments (68.5%). In 19 cases (35.2%), providers felt patients had not been offered all appropriate and available treatments, and in 9 cases (16.7%) providers encountered difficulties in finding such treatments for patients.

### Quantitative studies

The 5 key studies identified were systematic reviews and a survey of clinician practices and experiences with E/PAS in which questions related to the impact on the clinician were included. A review conducted by Ward et al. (2021) found 30 relevant papers across a broader time frame showing that healthcare practitioners experienced various emotional responses from providing E/PAS. Some HCPs “experienced positive emotions through helping patients at the end of the patient’s life,” while “other HCPs experienced very intense and negative emotions such as immense internal moral conflict” (Ward et al. 2021, 750). Additionally, a study by Rutherford et al. (2021) that reviewed 26 publications described an overarching theme of “tensions between palliative care and VAD.” Furthermore, Bellolio and Rosso (2021) conducted a systematic review which revealed that “available studies indicate that the decriminalisation of euthanasia has a negative impact on the professional work of physicians.” The study indicated further that in Flanders and the Netherlands, the physicians most affected are those who practice “primary or general medicine” since they are the primary recipients of E/PAS requests (Bellolio and Rosso 2021).

In 2020, Kelly and colleagues reported on their review of 9 studies with a specific focus on the impact on, or response from, physicians to their participation in E/PAS. According to their synthesis of research findings, where studies measured psychological impact, “30–50% of doctors described emotional burden or discomfort about participation,” while findings also identified “a comfort or satisfaction in believing the request of the patient was met” (Kelly et al. 2020, 86). Significant, ongoing adverse personal impact was reported for between 15% and 20%. The study noted that E/PAS can have a significant negative emotional impact on clinicians and despite the importance of the issue to medical practice, it is a largely neglected area of empirical research; “the limited studies to date highlight the need to address the responses and impact on clinicians, and the support for clinicians as they navigate this challenging area” (Kelly et al. 2020, 1).

### Qualitative studies

Twenty-two qualitative studies and 3 using mixed methods met accepted qualitative research assessment criteria (Hannes 2011). The following 5 themes arose from the synthesis of qualitative studies: (1) experience of the request prior to administration; (2) the doctor’s role and agency in the death of a patient; (3) moral distress post-administration; (4) increased workload and burnout; and (5) professional guidance and support.

#### Experience of the request prior to administration

For clinicians, the experience of a request for E/PAS can be varied and is often influenced by factors such as: the patient’s reason for the request, the response of the patient’s family to the request, and the physician’s own attitude towards E/PAS. According to de Boer et al. (2019), “The majority of Dutch physicians feel pressure when dealing with a request for euthanasia or physician-assisted suicide.” In this study, 6 categories of pressure were identified by Dutch General Practitioners including “emotional blackmail,” in reference to attempts by patients, families, and other colleagues to persuade practitioners to assent to E/PAS against their own conscience, control and direction by others, doubts about fulfilling criteria, counterpressure by patient’s relatives, time pressure around referred patients, and organizational pressure (de Boer et al. 2019). However, Ward et al. (2021), reported “all 8 physicians found providing MAiD to be very rewarding, despite the logistical challenges,” also adding that, “This study was limited to inquiring into the experiences of 8 physicians who were early adopters of MAiD and the findings are not generalizable to the experiences of other physicians” (Shaw et al. 2018). Clinicians also note that the support of the patient’s family for the E/PAS request is important. A participant from a study by Van den Ende et al. (2022) states:

I cannot imagine performing euthanasia on a patient whose wife does not support it. I cannot imagine anything like that. Then I would think we’d just have to put it off for a few weeks and we’d have to talk about it again. (D8, 4)

For the participants, a good death means including the family to participate in the last period of the patient’s life and not dying in solitude. This is in line with the findings of Ten Cate et al. (2017) who stressed the importance for physicians of “acceptance and resignation, being supported by loved ones, harmony, and being at home.” If the family had difficulty in accepting the approaching death of the patient, participants tried to take the time to get the family on board in order to create a safe and peaceful environment among loved ones for the patient in their final stages.

Regarding the physician’s own experience of the request – independent of the patient and family – the responses were varied. According to Ward et al. (2021), “Some HCPs experienced positive emotions through helping patients at the end of the patient’s life. Still other HCPs experienced very intense and negative emotions such as immense internal moral conflict.” Participants experienced conflict in balancing the desire to relieve the patient’s suffering and to do no harm, while balancing their own professional and personal duties.

#### “Being a doctor”: Role and agency in the death of a patient

The available studies indicate that clinicians can struggle to reconcile their role and agency in the death of a patient, especially when there are competing desires and requests between patients and families and their own conscience. Kelly et al. (2020) noted that “Participation in E/PAS can have a significant emotional impact on participating clinicians. For some doctors, participation can contrast with perception of professional roles, responsibilities, and personal expectations” (87). Furthermore, Gamondi et al. (2019) reported that “Many participants struggled to reconcile their understanding of palliative care principles with patients’ wishes to exercise their autonomy.” Similarly, according to Mathews and colleagues (2021), physicians had concerns about the “consumption of palliative care resources to support assisted death.” This originated from a fear that funding and resources will be taken from palliative care and poured into E/PAS reducing the quality of end-of-life care and increasing requests for E/PAS.

There were also concerns about doctors being seen as “providers” rather than people, leading to reduced physician autonomy and a lack of regard for the moral compass of the doctor. Although patient autonomy is crucial to good health care, participants expressed a desire for the impact on clinicians to be acknowledged simultaneously. As stated by Bellolio and Rosso (2021), “The acceptance to perform [E/PAS] has a significant emotional burden for the physician who must assume the role of executor of the patient’s will.” Similarly, in van Zwol et al. (2022), some participants indicated that they physically could not perform E/PAS until they believed it was justified in each situation:

I said: ‘Well, I am not ready yet.’ And then we discussed it again, and again, and finally I thought, now I find it palpable. Then I can go along with it. (D7, 4)

This study shows that physicians need room to act in accordance with their conscience and this may require time and consideration; “the participants were aware that euthanasia entails killing a human being, which made them reluctant to simply go along with the patient’s request” (van Zwol et al. 2022). However, since physicians are trained within the model of patient-centered care, staying true to their own conscience – a necessity for their own mental well-being – can be seen as selfish and a denial of the patient’s own needs, leading to feelings of guilt and shame. A participant from a study by Shaw et al. (2018) states:

Saying no to patients does not ever get easier…It still feels like the worst part of this work because those patients have come seeking help and as a physician, I very much want to help them. (398)

The conflict and tension between the physician’s own autonomy and that of the patient can cause significant moral distress for clinicians.

#### Moral distress post-administration

Studies reported that a significant proportion – over 50% – of physicians who have engaged in E/PAS experience moral distress, which can have long-term negative consequences on both individuals and the medical profession as a whole (Čartolovni et al. 2021). As Dholakia et al. (2022) noted, “Historically, medicine as a profession is rooted in the ethical principle of ‘first, do no harm’ while providing care”; due to this, a considerable number of participants responded that they experienced “polarised emotions including moral distress (*n* = 153).” This ethical principle of non-maleficence is not a social construct but rather an “intrinsic good,” held as a deeply rooted value in the human psyche (van Zwol et al. 2022). A participant from a study by Koksvik (2020) states:

I’m not an anxious person, I’ve been involved in lots of stuff, I’ve seen lots of people die and I felt weird. I felt, ‘gees, I’ve just killed somebody.’ (Physician 2, 6)

Similarly, participants from a study by Van den Ende et al. (2022) also shared their feelings about administering E/PAS:

To respect life. Yes, not because of faith. I mean, we all have the urge to live, we all want to live and not to die. (4)

My first reaction [to an E/PAS request] is always a fright. (5)

The first participant describes the incongruence of E/PAS with the usual human instinct that is the “urge to live.” Thus, some studies found that participants believed in working towards the development of palliative care and more adequate pain relief that would improve or restore an individual’s will to live rather than ending or giving up on the life of the patient (Lamb et al. 2022; van Zwol et al. 2022):

A patient may believe that continuing life is not worthwhile anymore because one suffers too much, experiences hopelessness and feels not being able to contribute to others (6).

On the other hand, some physicians believe that “ending suffering and fostering a good death, can override the moral duty to preserve life (van Zwol et al. 2022). This feeling was held by participants who felt they were unable to provide any more care to alleviate the suffering of patients and that E/PAS was the only possible solution. However, Wang (2017) notes that “Occasionally, the dying process is interrupted as a result of incomplete ingestion or vomiting of medications, confusion about timing of dying trajectory, familial emotional distress, and other variables.” In these cases, it is doubly distressing for physicians who already experience conflict regarding E/PAS but who have made the decision to alleviate suffering and allow patients to die comfortably; in these cases, it only exacerbates the pain for the patient and their family (Wang 2017).

St Ledger et al. (2021) reported that most instances of moral distress were “triggered by perceived futility, lack of continuity, protracted decisions, and failure to ensure ‘good death..’constraint[s] of clinical autonomy.” The study also noted that “moral distress was far reaching, affecting personal life, working relationships and career choice.” Physicians appear to be torn between wanting to preserve life and wanting to deliver “patient-centered” care: “she [the physician] is opposed to prescribing death-hastening medication but does not want to abandon her patient” (Brassfield et al. 2019). Patient-centered care and autonomy can often be in conflict with the professional responsibility and role of the physician.

#### Increased workload and burnout

Akin to the detrimental effect of moral distress on physicians is the experience of burnout. There is a strong correlation between moral distress and burnout as the manifestations and consequences are often similar, although burnout is associated more with mental and physical exhaustion than moral conflict (de Boer et al. 2019). The Maslach Burnout Inventory: Human Services Survey measures burnout in health professionals. According to Baigent and Baigent (2018), 3 main domains quantify the concept: exhaustion, cynicism (role negativity, feeling callous and detached), and professional efficacy (self-evaluation of competence and achievement). According to Khoshnood et al. (2018), physicians suggested that “the increase in workload related to MAiD is unsustainable.” Similarly, another study by Koksvik (2020) offered personal testimonies from physicians:

That [E/PAS pr MAiD] was very heavy because it meant that, you know … it’s terrible … like for instance, recently I had to do 2 interventions of MAiD in 24 hours. That’s too much. (Physician 3, 6)

I wouldn’t like to do a lot. I wouldn’t be able to do one or two a week, it’s too difficult for me. So, once in a while is okay, to help a patient, but not that much. (Physician 4, 6)

Participants who described experiencing burnout were often physicians who wanted to administer E/PAS to patients but found that the workload was unsustainable. One provider from a study by Beuthin et al. (2020) shared:

There were times when I was getting five or six referrals in a week, and it was completely overwhelming. (6)

In order to manage the excessive workload, physicians were often required to compensate by sacrificing family and personal time, working long hours, and relying on support from colleagues (Khoshnood et al. 2018):

I’m married with two children and the time that it’s taken from them has been hard on me. (Physician 17, 226)

I have been working 11-15 hours a day, not just on MAiD but because MAiD comes alongside and on top of my hospital and family practice. (Physician 8, 226)

Participants in the aforementioned studies have alluded to the significant pressure and demands placed on them on top of their already busy professional and family lives. The unsustainable hours and requests are leading the small number of E/PAS or MAiD providers to reconsider whether it is possible for them to continue engaging in this practice.

#### Professional support and relationship with colleagues

Professional support for physicians who have administered E/PAS is an important but neglected area of research according to the available studies. According to Kelly and colleagues (2020), despite 80% of the 245 physicians who granted a request of E/PAS reporting “feelings of discomfort: 49.6% of the physicians experienced it as burdensome, 45.8% as a heavy responsibility and 44.2% as emotional,” “the majority of the physicians (62.2%) did not seek support afterwards.” Another study by Evenblij et al. (2019) stated that physicians who do seek support after granting a request for E/PAS, support is sought from colleagues or relatives. Further explorations are required to “support policy decisions such as access to better emotional supports for providers and interdisciplinary support (Bouthiller and Opatrny 2019).

Relationships with colleagues is also a crucial aspect to physician well-being and career progression. This includes both doctors who perform E/PAS and those who desire to express a conscientious objection to E/PAS. According to McDougall and colleagues (2022), “For junior doctors wishing to exercise a conscientious objection to VAD, their dependence on their senior colleagues for career progression creates unique risks and burdens. In a context where senior colleagues are supportive of VAD, the junior doctors’ subordinate position in the medical hierarchy exposes them to potential significant harms: compromising their moral integrity by participating or compromising their career progression by objecting.” The evidence base indicates that support structures are lacking for both providers of E/PAS and those who choose to conscientiously object.

Furthermore, “little work has been done to understand and support healthcare professionals’ conscience. Instead, the rising polarity related to healthcare professionals’ freedom of conscience stems from a central lack of understanding of what conscience is and the relevance it holds for healthcare professionals’ clinical practice” (Lamb et al. 2022).

## Discussion

### Key findings

This review examined findings demonstrating the impact of administering E/PAS on clinicians. Identified publications included both survey-based quantitative data and qualitative research. The findings of this review confirm the findings of Kelly et al. (2020) as 21 out of the 30 studies report the presence of physician experience of psychological or moral distress. According to Wibisono et al. (2022), 52% of participants showed moral distress in response to an assisted death request from patients. In some instances, discomfort was related to an enduring perception by participants that, on reflection, not everything had been done to address the patient’s suffering and that requests were an indication that palliative care needed to be improved. This is exemplified in Anderson et al. (2022) in reference to some patient requests for E/PAS: “it was clear that their current advance directive did NOT accurately reflect their wishes, and was instead reflective of a poor-quality discussion, and fear around the lack of quality palliative care at the end of their life” (3). These concerns need to be studied and explored further to determine the best outcomes for patient comfort and physician mental health.

Physicians expressed concerns regarding a role change from being “an involved caregiver to being the mere provider of [E/PAS]” (Snijdewind et al. 2018). Furthermore, participants said that requests for E/PAS based on non-medical reasons came up more frequently and wondered if it was the right solution for these requests and that “the standards of E/PAS are shifting and that the boundaries of E/PAS regulation were stretched” (Snijdewind et al. 2018) Another fear held by participants was “an additional factor driving the request for E/PAS is demoralization, a state distinguishable from depression, that is associated with feelings of greater dependency on others or the perception of being a burden, along with existential distress, and a loss of meaning” (Kissane 2018). Studies of the last 6 years have demonstrated concerns with the challenge of psycho-existential distress as a source of suffering prompting requests for E/PAS that are distinguishable from the physical illness itself. These findings show that the patient’s reason or intention in seeking E/PAS matters and influences how clinicians perceive their role in the intervention.

The doctors who have participated in the reviewed studies want to treat patients in the best possible manner and help them die well. However, despite a significant proportion of doctors feeling rewarded by their involvement with E/PAS, “the available studies indicate that the decriminalization of euthanasia has a negative impact on the professional work of [many] physicians” (Bellolio and Rosso 2021). A study by Gamondi et al. (2019) showed that, “Many participants struggled to reconcile their understanding of palliative care principles with patients’ wishes to exercise their autonomy.” It is clear from the available literature that most physicians do not wish to exercise their authority in a paternalistic way. However, the studies indicate that there is often a genuine tension between what the doctor believes is best for the patient, what the family of the patient expect and what the patient desires. When this tension involves decisions at end of life, and specifically E/PAS, there can be detrimental effects on the psychological health and well-being of the doctor. Studies in this area have grown in the past decade; however, there is a need for more research and training in this area to provide better support for doctors experiencing moral distress in relation to E/PAS requests.

### What this study adds (strengths of the study)

Within the available literature, noteworthy findings emerged from this review. They suggest a substantial short- and long-term emotional impact (moral distress and moral injury) for a sizable proportion – over 50% – of clinicians who have received requests and participated in E/PAS (Wibisono et al. 2022). Personal and professional support needs are often unaddressed, with only a minority of those reporting adverse impacts seeking support from colleagues, and when support was sought, respondents relied chiefly on family or friends (Evenblij et al. 2019; Kelly et al. 2020).

### Limitations of the study and this field of research

All studies carry the important caveat of retrospectivity and varying time frames since participation in E/PAS. Bias is relevant in a topic that has been polarizing in both professional and public debates. Recruitment and participation biases need to be considered, as it is possible that those most affected by their experience will be most motivated to participate in the studies. The selected papers were reviewed in detail by one of the authors (PJP), and the authorship team was comprised of both clinical and discipline expertise and perspectives: oncology (GBF) and psychiatry and palliative care (DWK). Quantitative findings can be limited, being exploratory and based on limited items within larger surveys of clinical practice. Finally, this review focuses on physicians, however, nurses are being asked in some jurisdictions to deliver E/PAS and future reviews might focus on moral distress among them. Given these limitations in the research conducted to date, it would be unwise to generalize to disciplines outside medical practitioners, and allow for the exploratory thematic nature of qualitative research.

## Conclusion

Participation in E/PAS has been shown to be associated with moral distress in over 50% of clinicians (Wibisono et al. 2022). For many clinicians, this can include significant adverse long-term personal and professional consequences, thereby impacting the medical profession as a whole. The experience of providing E/PAS has led some physicians to reflect on how palliative care may be improved for patients and their families, encompassing the “psychiatric, psychosocial, existential, and spiritual aspects of care” (Breitbart 2012, 153). While others have had the sense of having respected a patient’s last wishes and granted what they believe to be a compassionate death.

These findings showcase the complexity of E/PAS for participating clinicians, highlighting the need for further research into the impact of administering E/PAS on doctors specifically focusing on moral injury; better support structures for doctors post-administration; greater funding and strategies to improve palliative care for patients who seek E/PAS due to a sense of demoralization; and how to understand why some doctors are negatively emotionally affected by involvement in E/PAS, while others can feel fulfilled by similar actions. How do we adequately support clinicians to navigate this challenging area? Furthermore, another question to be posed is: is it necessary to implicate doctors in E/PAS? Much of the conflict arises from the fact that medical professionals are trained to save and preserve life and asking clinicians to do the opposite may be a rectifiable injustice. According to Preston et al. (2023), “Several countries have adopted de-medicalized approaches. In Switzerland assisted dying is considered a civil rather than a medical act.” This reduces the burden of responsibility on doctors, and they “could instead focus on becoming more confident in having compassionate conversations when responding to requests for assisted dying” (Preston et al. 2023, 1). Evidently, legally practiced assisted dying is an ethically complex area in need of further empirical and conceptual work.
